# On Stone in the Female Bladder

**Published:** 1885-04-20

**Authors:** George H. Kidd

**Affiliations:** M. D. Edin., President of the Obstetric Section of the Academy of Medicine, Ireland


### ON STONE IN THE FEMALE BLADDER.

BY GEORGE H. KIDD, M. D. EDIN.,

**President of the Obstetric Section of the Academy of Medicine, Ireland.**

Stone in the bladder of women, though of rather infrequent occurrence, is met with sufficiently often to make it incumbent on the surgeon to sound the bladder in all cases where there may be anything in the symptoms indicative of vesical trouble. No doubt the bladder is constantly affected sympathetically in cases of disease of the urethra, vagina, uterus, kidneys, and rectum, and their surrounding parts; but sounding the female bladder is easily done, occupies but a few seconds, causes little or no pain, and leaves no injurious results. If no stone be found, attention can then be directed more definitely to other organs; and if one be found, the true nature of the case is at once established. The ordinary uterine sound suits perfectly for exploring the female bladder. It can be used during a vaginal examination—the patient lying on the side, in the usual obstetric position—without creating any alarm, or indeed anything being said on the subject. In doubtful cases, where no stone is detected, and the symptoms still tend to the suspicion that there may be one present, it is well to place the patient on her back, and if the bladder be empty inject water into it, and again pass the sound, but this is rarely necessary.

There are two methods of removing a stone from the female bladder—through the urethra, or through an opening made in the base of the bladder from the vagina. Many modern surgeons prefer the method by incision—Marion Sims and Emmet have both recommended it. The success that has in modern times attended operations for vesico-vaginal fistula has had much influence in deciding surgeons to adopt this method, along with the apprehension of incontinence of urine as a consequence of dilatation of the urethra. Two forms of lithotomy have been recommended—1st, through the vesico-vaginal septum; 2nd, through the urethra, laying it open for about 1½ inches behind the meatus, and running the incision, if the size of the stone require it, into the bladder. The first is certainly the better of these operations. The sphincter should never be touched if it can by any possibility be avoided. Emmet specially recommends incision through the vesico-vaginal septum where there is chronic cystitis to any extent. In such cases he directs that the opening should not be closed after the stone has been extracted, but left to form a fistula, so as to leave the bladder at rest till the mucous membrane recovers its healthy condition. The success of the method of treating chronic cystitis by the establishment of a fistula lends much weight to this recommendation in aggravated cases of cystitis, but as a rule, the lining membrane of the bladder gets into healthy condition very rapidly after the stone has been removed, and the establishment of a fistula is very rarely necessary.

Dilatation of the urethra is the oldest method of removing calculi from the female bladder. It is objected to because of the risk of incontinence of urine, but as practiced of late the risk of this is really very little. I have dilated the urethra very frequently, and am not at all sure that in any case permanent incontinence followed in consequence.

All modern surgeons who operate by the urethra prefer rapid dilatation without incision. This may be done with Weiss' instrument or with the finger, the patient being under the influence of an anæsthetic. Weiss' instrument may be passed into the bladder and dilated rapidly, but the edges of the arms are liable, when expanded, to cut the mucous membrane.

To obviate this Mr. Bryant covers them with india-rubber, but dilatation with the finger, as recommended by Mr. Christopher Heath, has always appeared to me a safer and better operation. The patient being brought under the influence of an anæsthetic, is placed in the lithotomy position, and the hands and feet tied together, then, according to Mr. Heath, a director is pressed into the bladder. I prefer a probe, believing the edges of a grooved director are liable to cut the mucous membrane. The finger is then gradually passed into the bladder by a semi-rotary motion, the probe or director being used as a guide, so as to avoid making a false passage. A large sized bougie may be passed first, then the little finger, and afterward the index-finger.

A few minutes will suffice to do this. The size and character of the stone may now be ascertained. If it be not very large, the lithotomy forceps may be passed in, the stone seized, and withdrawn, its position having been fixed by the finger, so that it could be seized in its shortest diameter. If the stone be too large, then it must be broken down, and for this purpose the crusher devised by Mr. Erichsen for breaking the stone after lithotomy in the male or an instrument of that description, answers better than the lithotrite. It can be passed in the same way as the forceps, the position of the stone having been clearly ascertained by the finger, and the stone seized and broken. If necessary, a finger in the vagina will aid in the seizing of it. If the stone consists of tripple phosphates, as large stones usually do, it will break down with great ease. Whether a mulberry calculus could be broken in this way, I cannot say; but they are seldom so large as to require to be broken. After breaking the stone and removing all the fragments with the forceps and the finger, the bladder should be washed out, which may be done with an ordinary catheter and syringe.—*Dublin Journal of Medical Science, Sept., 1884, p. 197. Braithwaite.*

